# β3-adrenergic receptor agonist causes acute thermogenic metabolic crisis in ACSS1-K635Q knock-in mice

**DOI:** 10.7150/ijbs.122643

**Published:** 2026-01-01

**Authors:** E. Sandra Chocron, David Zhang, Bushra Sumawi, Joseph Schell, Diego Cruz, Guannan Li, Prethish Sreenivas, Haiyan Jiang, Felix F. Dong, Erin Munkácsy, Shangang Zhao, Maria A. Gonzalez Porras, David Gius

**Affiliations:** 1 Department of Radiation Oncology, Mays Cancer Center at UT Health San Antonio MD Anderson, Joe R. and Teresa Lozano Long School of Medicine, San Antonio, Texas, United States.; 2 Barshop Institute for Longevity and Aging Studies at UT Health San Antonio, San Antonio, Texas, United States.; 3 Department of Medicine, UT Health San Antonio, San Antonio, Texas, United States.; 4 Department of Biomedical Engineering and Chemical Engineering, The University of Texas at San Antonio, San Antonio, TX.

**Keywords:** ACSS1, acetylation, beige, brown adipose tissue, brown fat, mitochondria, thermogenesis, UCP1

## Abstract

Mitochondrial Acyl-Coenzyme Synthetase Short Chain Family Member-1 (ACSS1) converts free acetate into acetyl-coenzyme A (acetyl-CoA), regulated, in part, by acetylation at lysine 635 (ACSS1-K635). We challenged our ACSS1 constitutive acetylation mimic knock-in (K635Q) mice by injecting a β3-adrenergic receptor agonist, CL-316243 (CL), to induce a thermogenic response. Strikingly, we show that *Acss1^K635Q/K635Q^* mice exhibit hypothermia and acute metabolic crisis following CL stimulus, as shown by significantly reduced oxygen consumption, carbon dioxide production, respiratory exchange ratio, and heat production. We also observed histological differences in both brown adipose tissue (BAT) and subcutaneous white adipose tissue (WAT), accompanied by altered expression and regulation of lipogenic enzymes and Uncoupling Protein 1 (UCP1) in *Acss1^K635Q/K635Q^*. In contrast to wild-type adipose tissues, *Acss1^K635Q/K635Q^* did not show changes in acetyl-CoA and acetate levels in response to CL, and mitochondria isolated from BAT displayed impaired respiration on palmitate. Lastly, beige adipocytes differentiated *ex vivo* from *Acss1^K635Q/K635Q^* mice showed altered response to the adenylate cyclase stimulator, forskolin, with unresponsive mitochondria and lipogenic lipid droplets, and lower fatty acid oxidation activity. These results suggest that non-acetylated ACSS1 plays an essential role in thermoregulation and the ability to metabolize free fatty acids.

## 1. Introduction

Acetyl-Coenzyme A (acetyl-CoA) is essential to energy metabolism and lipid synthesis, as well as epigenetic and protein regulation 1, 2. In mammalian cells, the majority of acetyl-CoA is generated through glycolysis and fatty acid oxidation (FAO). As such, acetyl-CoA functions as a small molecule metabolic sensor and regulates protein function through non-enzymatic acetylation of lysine residues 3, 4. Thus, increased FAO, as under both fasting and high-fat diet conditions, increases acetylation of ACSS1 and other mitochondrial proteins 10, 11. Acetyl-CoA Synthetase Short Chain Family Member-1 (ACSS1, previously known as AceCS2) catalyzes the ligation of acetate and coenzyme A to generate acetyl-CoA in the mitochondria, and plays a role in lipid metabolism, at least under certain nutrient conditions 5. ACSS1 enzymatic activity is regulated, in part, through acetylation at lysine 635 in mice (K642 in humans), which reduces ACSS1 activity, and by deacetylation by Sirtuin 3 (SIRT3) 6-9.

As the primary mitochondrial deacetylase, SIRT3, plays an important role in regulating metabolism, including FAO 12 and brown fat thermogenesis 13. Brown adipose tissue (BAT) dissipates energy as heat during non-shivering thermogenesis. Studies in both humans and murine models have demonstrated that higher levels of brown fat are protective against developing type 2 diabetes and cardiovascular diseases 14-16. When stimulated by cold or thyroid hormone, BAT uses glucose and free fatty acids (FFAs) released from triglycerides by lipases for thermogenesis. Fully active thermogenic adipose tissue can increase whole-body energy expenditure by more than 100% in mice 17 and 40-80% in humans 18.

Importantly, white adipose tissue (WAT) can go through a browning process, with “beige” adipocytes appearing in WAT in response to increased thermogenic demand from cold stimulus or pharmaceuticals. Both beige and brown adipocytes contain multilocular lipid droplets and a high density of mitochondria that express Uncoupling Protein 1 (UCP1). When activated, UCP1 creates an alternate portal for protons back across the inner mitochondrial membrane, thereby uncoupling oxidative phosphorylation and generation of the proton gradient from ATP synthesis and dissipating the energy potential as heat instead 19. Adipocyte thermogenic capacity thus depends primarily on catabolism of FFAs through oxidative metabolism 20. Classically, adipose-tissue thermogenesis is driven by sympathetic nerve-mediated adrenergic signaling, which stimulates lipolysis. FFAs serve both as fuel for thermogenesis and as allosteric activators of UCP1 function. Indeed, long-chain fatty acids are uniquely required for UCP1-induced uncoupling 21. WAT is key in this process by releasing FFAs into circulation through lipogenesis to support BAT thermogenesis 22.

While acetylation has been shown to decrease ACSS1 ligase activity 7, the physiological role of ACSS1 acetylation status in thermogenesis has not been characterized. In this regard, we generated an ACSS1-acetylation (Ac) mimic knock-in mouse, where lysine 635 was mutated to glutamine (K635Q), which structurally and biochemically mimics acetylated lysine 5, 9. In metabolic conditions requiring increased reliance on FAO, including fasting 5 or on a ketogenic diet 23, these mice exhibited hepatic steatosis and altered lipid profile compared to control mice under the same conditions. These data suggested that *Acss1^K635Q/K635Q^* mice have difficulty with increases in metabolic demand that depend on FAO. For this reason, we investigated the role of ACSS1 acetylation in the thermogenic response, which is highly dependent on fatty acid metabolism, by injecting *Acss1^K635Q/K635Q^* mice with the specific β3-adrenergic receptor agonist, CL-316243 (CL). Strikingly, we found *Acss1^K635Q/K635Q^* mice to be intolerant of CL injection, exhibiting acute metabolic decompensation, hypothermia, and in some cases, death 24 hours after injection.

## 2. Methods

### 2.1. Animal model

All animal experiments were conducted in compliance with the National Institutes of Health (NIH) Guidelines for Humane Care and the Use of Laboratory Animals, and all murine studies were approved by the University of Texas Health San Antonio (UTHSA) Institutional Animal Care and Use Committee. Our *Acss1^K635Q^* mouse was generated in a C57BL/6 background and has been described in detail previously 5. Mice were maintained in specific pathogen-free conditions at 21 ± 2 °C with 12h:12h light:dark cycle in the Animal Facility at UTHSA. Mice had free access to food and water, and were given pelleted Inotiv Teklad Rodent Diet traditional formula LM-485. For all studies, we used four-month-old male and female mice, as indicated for each experiment. Confounders such as order of measurements and cage location were not controlled.

*Thermogenic stimulation:* CL-316243 (CL) was prepared in saline at a solution of 0.2 mg/mL and then injected in mice intraperitoneally at 1 mg/kg. Then mice were monitored every hour for measurements and health status hourly. Temperature was measured using a mouse rectal thermometer. A Countour Next EZ glucometer and glucose strips were used to measure blood glucose from a small tail snip. Rectal temperature was monitored at the time points indicated with a rodent rectal temperature probe Ret 3 (Kent scientific).

*Evaluation of circulating lipids*: Whole blood was collected from the mice and serum was obtained by letting the blood to clot for one hour and then spun 10 minutes at 2000xg. Supernatants were then stored minus 80. Serum was sent to IDEXX to analyze free fatty acids, triglycerides and cholesterol based on a rodent panel.

*Respirometry measurements*: Mice were individually housed in metabolic cages, with free access to food and water. Mice were acclimated for two days before recording baseline parameters with PhenoMaster (Sable Systems) and following injection with CL (1 mg/kg) the mice are used for measurements of respiration for the following 24 hours.

### 2.2. Histology

Tissue blocks of formalin-fixed, paraffin-embedded mouse tissues were processed by the Laboratory Medicine core, UT Health San Antonio using standard methods. When adequate color development was seen, slides were washed in water to stop the reaction, counterstained with Meyer's hematoxylin (Dako), and covered with a Permount mounting medium (Richard-Allan Scientific). The micrographs were taken under a light microscope (Leica). Representative images are shown.

### 2.3. Mitochondria isolation and oxygen consumption assays

Approximately 10 mg BAT was collected on ice for Seahorse analysis. Mitochondria were isolated from tissue as previously described. In brief, the tissue was placed in a mitochondrial isolation buffer [70 mM sucrose, 210 mM mannitol, 1 mM ethylene glycol tetraacetic acid (EGTA), 5 mM 4-(2-hydroxyethyl)-1-piperazineethanesulfonic acid (HEPES), pH 7.2] and homogenized using a glass Teflon Dounce homogenizer. This solution was then centrifuged twice at 1000 g for 5 min at 4 °C to remove cell debris. The mitochondrial pellet was resuspended in a mitochondria assay solution (MAS) (70 mM sucrose, 220 mM mannitol, 5 mM phosphate, 5 mM magnesium chloride, 1 mM EGTA, 2 mM HEPES, pH 7.4). Protein was quantified by bicinchoninic acid (BCA) assay (Pierce) and concentrations adjusted with MAS to 3 μg of protein. The Seahorse plate containing 100 μL/well of the mitochondrial suspensions was centrifuged at 2000 g for 5 min at 4 °C with free deceleration. Finally, the well volume was brought up to 180 μL by addition of MAS supplemented with malate. The oxygen consumption rate (OCR) was measured in the presence of 10 mM BSA-conjugated palmitate, using a Seahorse XFe96 analyzer at 37 °C, and 5 mM glycerol-3-phosphate (G3P) and 3 mM guanosine diphosphate (GDP) were added sequentially at controlled time points during the OCR measurements. Data were collected and processed using Agilent Seahorse Wave software.

### 2.4. Western Blotting and Imaging

Total protein lysate preparation from tissues for immunoblot have been described 24-27. Protein concentrations were determined by Pierce BCA Protein Assay, and 20 µg from each sample was separated by NuPAGE^TM^ Bis-Tris 4-12% gradient sodium dodecyl sulfate polyacrylamide gel electrophoresis (SDS-PAGE) (Invitrogen), transferred onto polyvinylidene difluoride (PVDF) membrane (Bio-Rad), probed with primary antibody (Table 1) and then with horseradish peroxidase (HRP) conjugated to either a mouse or rabbit secondary antibody (Cell Signaling), and visualized with enhanced chemiluminescence (Fisher Scientific; SuperSignal^TM^ West Femto Maximum Sensitivity Substrate).

Immunoblots were imaged were obtained using ProteinSimple FluorChem M, using the Auto Exposure feature to takes successively longer exposures of the membrane until an optimum exposure time is achieved. The subsequent images are then acquired in standard resolution, using 4x4 pixel binning (832x626-pixel images).

### 2.5. Quantitative real-time polymerase chain reaction analysis

Total RNA was extracted and purified from iWAT tissue and cells using the RNeasy Mini Kit (Qiagen), following the manufacturer's instructions. mRNA concentrations were determined with a Take3 Micro-Volume Plate (BioTek) and normalized to 150 ng prior to cDNA synthesis. Complementary DNA was generated using random hexamer primers and the iScript cDNA synthesis kit (Bio-Rad). Quantitative RT-PCR was carried out using the SsoAdvanced™ Universal SYBR Green Supermix (Bio-Rad) on a CFX96 Touch Real-Time PCR Detection System (Bio-Rad). Relative gene expression was quantified using the 2^-∆∆Ct^ method, with transcript levels normalized to 18S rRNA. Primer specificity was confirmed via melting curve analysis (see Table 2 for sequences).

### 2.6. Acetyl-Coenzyme A and Acetate Quantification

We used frozen BAT and iWAT to quantify acetyl-CoA and acetate using the Abcam fluorometric PicoProbe^TM^ acetyl-CoA (ab87546) and colorimetric acetate (ab204719) assay kits, respectively. Results were normalized to protein, as measured by Pierce BCA assay.

### 2.7. Cell Culture

*Isolation:* Subcutaneous adipose depots were harvested from euthanized WT and *Acss1^K635Q/K635Q^* mice and digested in collagenase type I. The adipose-derived stem cells were enzymatically isolated following a previously published protocol 28. Briefly, digestion was performed in an orbital shaker at 37 °C for 60 min. The digest was then centrifuged and the floating adipocyte layer discarded. The pelleted stromal vascular fraction was washed two times with complete media [Dulbecco's Modified Eagle Medium (DMEM) with Ham's F12 nutrient mixture, supplemented with 10% fetal bovine serum and 1% antibiotic-antimycotic] and plated on tissue culture plastic. Cells at passages 2 to 5 were used for experiments.

*Adipose derived stem cells beige differentiation:* Cells (passage 2-4) were cultured in 24-well tissue culture plates and incubated in complete media. When cells reached 95% confluence, differentiation was initiated. For beige differentiation, cells were incubated for 4 days in induction media (complete media with 5 μg/mL insulin, 10 μM forskolin, 2 μg/ml dexamethasone, 125 μM indomethacin, 0.5 μM rosiglitazone and 1 nM triiodothyronine) and then maintained in complete media with 5 μg/ml insulin, 10 μM forskolin, 1 μM rosiglitazone and 1 nM triiodothyronine. Differentiation media was changed every other day until harvest.

*Evaluation of fatty acid oxidation, lipid droplets, and mitochondrial membrane potential in mature adipocytes in vitro:* FAO Blue was used at 1 μM concentration in DMEM without serum or phenol red. Beige differentiated stromal vascular cells were incubated in this media for 15 minutes in a humidified incubator and then directly imaged with a Leica Stellaris Sp5 confocal with the 405 laser line. Images were acquired at 63x and then fluorescence intensities were analyzed with the LAX software. To assay lipid droplets and mitochondrial membrane potential, we used bodipy at 1 μM and tetramethylrhodamine methyl ester (TMRM) ratiometric dye at 10 nM, and incubated cells for 10 minutes in a humidified incubator before imaging at 488 and 563 excitation. The lipid droplets and mitochondria were analyzed with LAX software by binning for size, count, and fluorescence intensity.

### 2.8. Statistical Analysis

Densitometric analysis of protein quantitation was determined by ImageJ software v0.5.5. (National Institutes of Health; http://imagej.nih.gov/ij Java 1.8.0- internal). Statistical differences were calculated as indicated in the figure legends by one- or two-way ANOVA, multivariate analysis, or unpaired Student's *t*-test in Microsoft Excel v2501 or GraphPad Prism 10.4.1. *P*-values were two-sided, and tests were considered significant at *P* < 0.05. All measurements were taken from distinct samples and replicates are biological. No data points were excluded. The number of replicates (mice) for each experimental group is given in the figure legends in the main text and supplement.

## 3. Results

### 3.1. *Acss1^K635Q/K635Q^* mice are intolerant to β3-adrenergic receptor agonist

**Figure 1:**
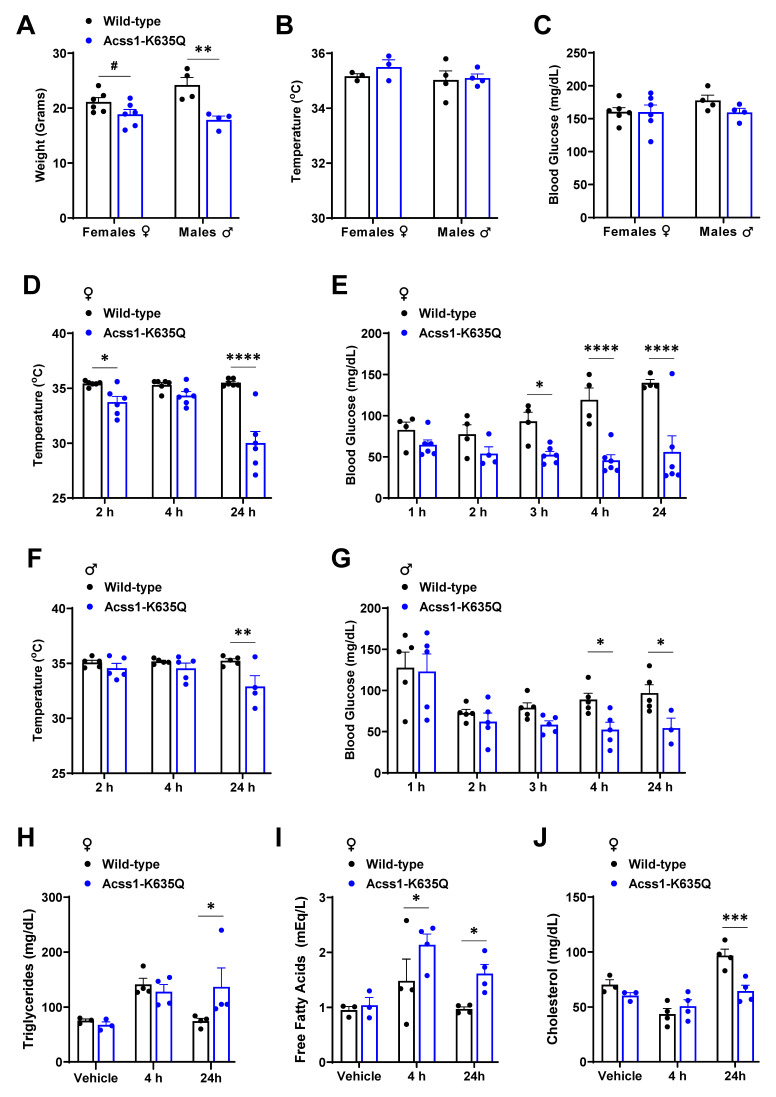
***Acss1^K635Q/K635Q^* mice show impaired response to β3 adrenergic agonist, CL-316243. (A-C)** Body mass (A), rectal temperature (B), and blood glucose (C) were measured in female and male 4-month-old WT and *Acss1^K635Q/K635Q^* mice. Exact data points (each representing an individual mouse) are shown, as well as the mean ± SEM. Statistical comparisons of WT vs. *Acss1^K635Q/K635Q^* were calculated for each sex and parameter by unpaired *t*-test (^#^*p* < 0.1, **p < 0.01). **(D-G)** At the indicated time points post-injection of CL-316243 (CL), rectal temperature (D, F) and blood glucose (E, G) were measured in female (D, E) and male (F, G) WT and *Acss1^K635Q/K635Q^* mice. Exact data points (each representing an individual mouse) are shown, as well as the mean ± SEM. Statistical comparisons of WT vs. *Acss1^K635Q/K635Q^* were calculated at each timepoint by two-way Analysis of Variance (ANOVA) with Tukey's Multiple Comparisons test (*p < 0.05, **p < 0.01, ****p < 0.0001). **(H-J)** Serum triglycerides (H), non-esterified free fatty acids (I), and cholesterol (J) were measured in female WT and *Acss1^K635Q/K635Q^* mice given vehicle vs. 4- or 24-hours post CL injection. Exact data points (each representing an individual mouse) are shown, as well as the mean ± SEM. Statistical comparisons of WT vs. *Acss1^K635Q/K635Q^* were calculated at each timepoint by two-way ANOVA with Tukey's Multiple Comparisons test (*p < 0.05, ***p < 0.001).

As we have previously demonstrated, *Acss1^K635Q/K635Q^* mice are phenotypically normal, except for a notably smaller body mass in males 5. Here, we show this size difference continues to be significant at three months on standard chow fed *ad libitum* (Figure 1A). We found no difference in body temperature or blood glucose levels in either sex (Figure 1B,C). Our previous studies showed that *Acss1^K635Q/K635Q^* mice seem to have impaired lipid metabolism and develop fatty liver under nutrient conditions demanding greater reliance on FAO, such as fasting 5 and a ketogenic diet 24. Here, we turn our focus from the liver to adipose tissue, and test the thermogenic response in *Acss1^K635Q/K635Q^* mice.

To induce a thermogenic response, three-month-old mice were administered the potent selective β3-adrenergic receptor agonist, CL-316243 (CL), by intraperitoneal (IP) injection at 1 mg/kg. Rectal temperature was monitored every 2 hours and blood glucose hourly. Both male and female *Acss1^K635Q/K635Q^* mice exhibited a drop in body temperature and blood glucose levels that was greatest after 24 hours, and more significant in female mice (Figure 1D-G), compared to no change in WT mice. In addition, roughly 25% of *Acss1^K635Q/K635Q^* mice died 24 hours after injection. These results demonstrate *Acss1^K635Q/K635Q^* mice are acutely intolerant to β3-adrenergic stimulation by CL and unable to maintain blood glucose and proper thermogenic function. In addition, these data further suggest that these mice are vulnerable to conditions that require increased fatty acid metabolism.

We further characterized the response to CL in female mice since they displayed more severe effects. In this regard, serum lipids were measured in mice given saline versus 4- and 24-hours post CL injection. We saw an increase in serum triglycerides 4 hours post-injection in both wild-type (WT) and *Acss1^K635Q/K635Q^* mice, with minimal difference between genotypes (Figure 1H). In contrast, *Acss1^K635Q/K635Q^* mice showed a significant increase in circulating FFA post-injection (Figure 1I), suggesting that CL stimulates lipolysis but that *Acss1^K635Q/K635Q^* mice are unable to effectively utilize circulating FFA. While cholesterol levels followed a similar trend in both genotypes, the dip and subsequent increase in WT post-injection appeared to be of somewhat greater magnitude (Figure 1J).

### 3.2. *Acss1^K635Q/K635Q^* mice are unable to adapt to the metabolic demand of CL stimulation

**Figure 2:**
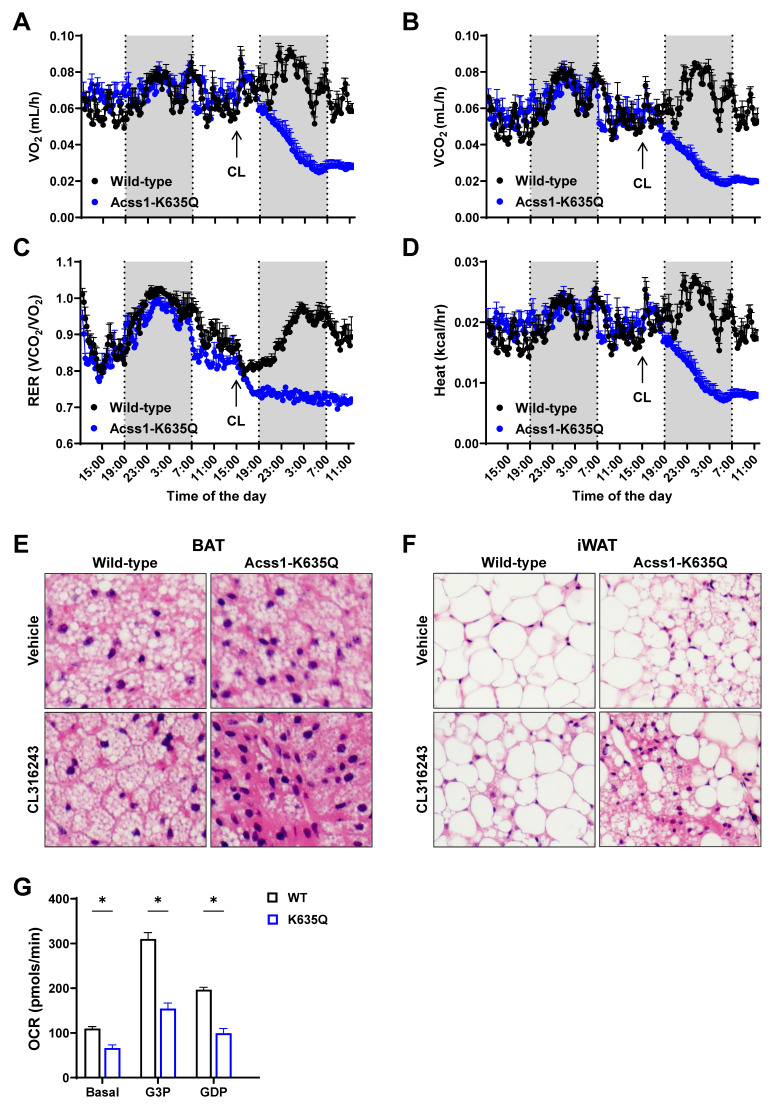
***Acss1^K635Q/K635Q^* mice are unable to adapt to the metabolic demand of CL stimulation. (A-D)** Individual metabolic cages were used to measure oxygen consumption rate (A), carbon dioxide production (B), respiratory exchange ratio (C), and heat production (D), normalized to body mass, in 3-month-old female WT and *Acss1^K635Q/K635Q^* mice (*n* = 4 mice per group). Data represented as mean ± SEM at each timepoint. **(E)** H&E staining of brown adipose tissue (BAT) collected from WT (left column) and *Acss1^K635Q/K635Q^* (right column) mice 24 hours after injection of vehicle (top row) or CL (bottom row), shown at 40x magnification (*n* = 3 mice per group). 10x magnification is shown in Supplemental Figure S1B. **(F)** H&E staining of inguinal white adipose tissue (iWAT) collected from WT (left column) and *Acss1^K635Q/K635Q^* (right column) mice 24 hours after injection of vehicle (top row) or CL (bottom row), shown at 40x magnification (*n* = 3 mice per group). 10x magnification is shown in Supplemental Figure S1C. **(G)** Oxygen consumption rate of mitochondria isolated from BAT of 3-month-old female WT and *Acss1^K635Q/K635Q^* mice sequentially treated with 5 mM G3P and 3 mM GDP (*n* = 2 mice per group with 6 technical replicates each). Data shown as mean ± SEM. Comparisons were made by unpaired *t*-test (*p < 0.05).

To further characterize the metabolic response of three-month-old females to CL stimulation, we performed respirometry measurements in individualized cages. While acclimating the mice for two days, we did not see major metabolic differences as we had previously in male mice 5. However, following CL injection, we saw dramatic effects in the metabolic parameters of *Acss1^K635Q/K635Q^* mice. While WT mice responded to CL with increased O_2_ consumption (Figure 2A), *Acss1^K635Q/K635Q^* mice showed a dramatic decrease in O_2_ consumption, CO_2_ production, respiratory exchange ratio, and heat (Figure 2A-D), which may play a mechanistic role in the 25% lethality observed 24 hours post-injection. These data clearly demonstrate that *Acss1^K635Q/K635Q^* mice are intolerant to the thermogenic stimulus elicited by CL, suggesting a potential acute disruption of metabolism.

We collected subcutaneous inguinal WAT (iWAT) and BAT at 0, 4, and 24 hours after CL injection to evaluate tissue histology and protein expression levels. We observed that, following injection, the suprascapular subcutaneous WAT depot became less apparent in WT mice but remained unchanged in* Acss1^K635Q/K635Q^* (Suppl. Figure 1A), suggesting that *Acss1^K635Q/K635Q^* mice were unable to utilize lipids from WAT depots during thermogenic stimulation.

Hematoxolin and eosin (H&E) staining showed a homogenous distribution of smaller lipid droplets in BAT from WT mice 24 hours after CL injection (Figure 2E, bottom left panel, and Suppl. Figure 1B). In contrast, BAT from *Acss1^K635Q/K635Q^* mice exhibited disorganized lipid droplet distribution with some cells containing large lipid droplets while others appeared completely exhausted with areas of dark staining (Figure 2E, bottom right panel, and Suppl. Figure 1B). H&E staining revealed a similar trend in iWAT, with *Acss1^K635Q/K635Q^* showing a heterogenous distribution of lipid droplets (more cells with smaller lipid droplets) as well as multilocular cells, which are not normally found in this tissue under basal conditions (Figure 2F, upper panels). After CL injection, iWAT from WT mice displayed smaller adipocytes and some multilocular cells (Figure 2F, lower left), while *Acss1^K635Q/K635Q^* showed numerous multilocular cells and areas of lipid exhaustion (Figure 2F, lower right panel, and Suppl. Figure 1C). These results imply a disruption of the FFA mobilization required for the thermogenic response and suggests that *Acss1^K635Q/K635Q^* mice are unable to metabolically adapt to the acute thermogenic response elicited by CL.

We also performed seahorse oxygen consumption assays with mitochondria isolated from WT and *Acss1^K635Q/K635Q^* BAT. In the presence of 10 mM BSA-conjugated palmitate, *Acss1^K635Q/K635Q^* BAT mitochondria showed lower basal respiration and reduced response to glycerol-3-phosphate (G3P), while inhibiting UCP1 with guanidine diphosphate (GDP) reduced oxygen consumption rate (OCR) by approximately 64% in both genotypes (Figure 2G). These results further suggest that mitochondria from *Acss1^K635Q/K635Q^* BAT show deficient respiration in the presence of palmitate, pointing out to deficient FAO metabolism which may impair thermogenesis.

### 3.3. *Acss1^K635Q^* adipose tissue displays dysregulated lipogenic signaling

**Figure 3:**
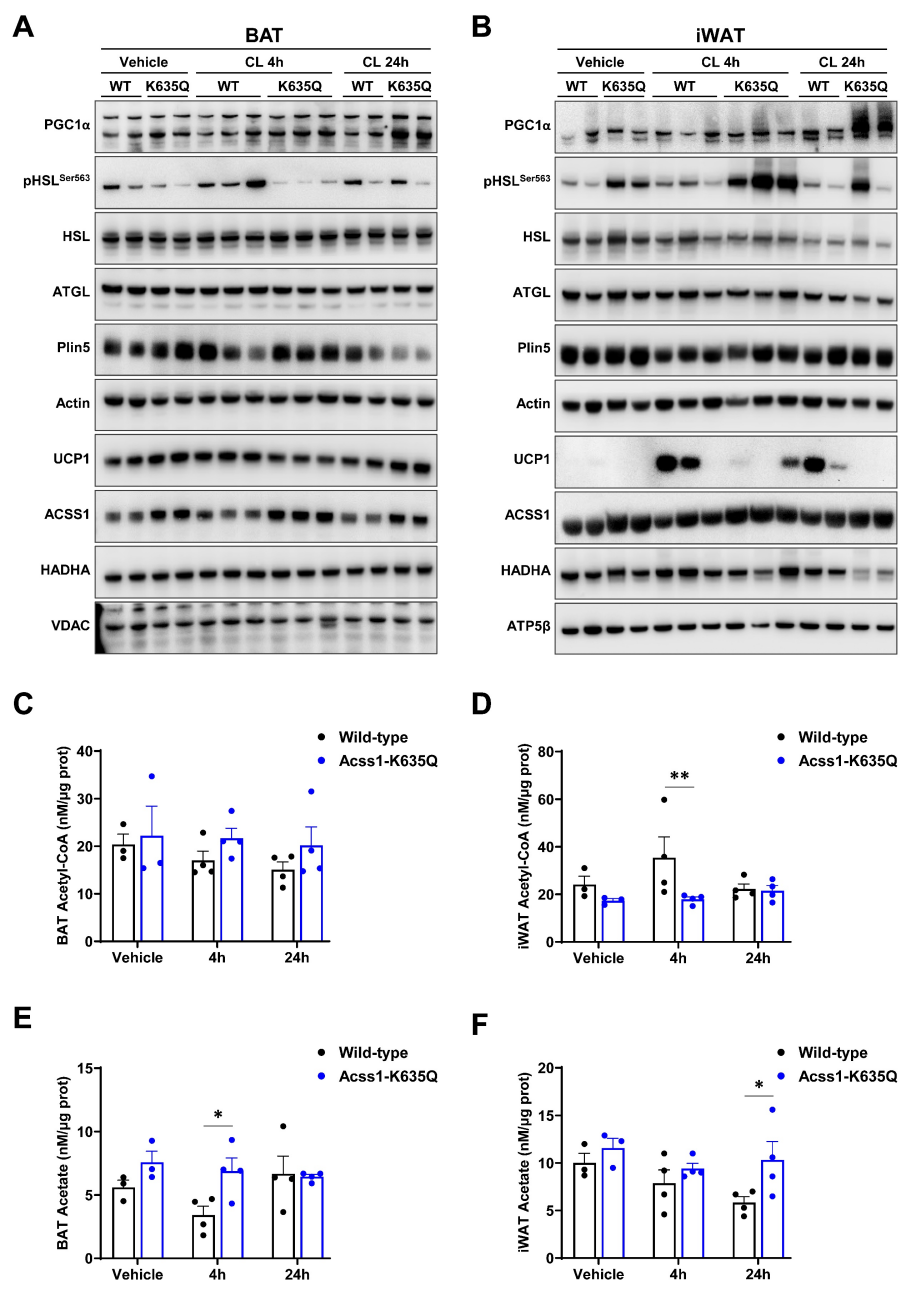
** Acss1-K635Q alters fatty acid utilization upon β3 adrenergic stimulation. (A)** Immunoblots show protein levels in WT and *Acss1^K635Q/K635Q^* BAT following vehicle and 4 and 24 h CL treatment. VDAC serves as loading control for mitochondrial proteins UCP1, ACSS1, and HADHA (*n* = 3 mice for vehicle and 4 per treatment group). Quantification is shown in Supplemental Figure S2. **(B)** Immunoblots show protein levels in WT and *Acss1^K635Q/K635Q^* iWAT following vehicle and 4 and 24 h CL treatment. ATP5β serves as loading control for mitochondrial proteins UCP1, ACSS1, and HADHA (*n* = 3 mice for vehicle and 4 per treatment group). Quantification is shown in Supplemental Figure S3. **(C-D)** Acetyl-CoA levels were measured in WT and *Acss1^K635Q/K635Q^* BAT (C) and iWAT (D), and normalized to protein levels. Exact data points (each representing an individual mouse) are shown, as well as the mean ± SEM. Statistical comparisons of WT vs. *Acss1^K635Q/K635Q^* were calculated at each timepoint by two-way ANOVA with Tukey's Multiple Comparisons test (**p < 0.01). **(E-F)** Acetate levels were measured in WT and *Acss1^K635Q/K635Q^* BAT (E) and iWAT (F), and normalized to protein levels. Exact data points (each representing an individual mouse) are shown, as well as the mean ± SEM. Statistical comparisons of WT vs. *Acss1^K635Q/K635Q^* were calculated at each timepoint by two-way ANOVA with Tukey's Multiple Comparisons test (*p < 0.05).

Immunoblots were done to assess relative levels of major lipogenic, lipolytic, mitochondrial biogenesis, and thermogenic response proteins in BAT and iWAT at baseline and following CL injection (Figure 3A,B, quantified in Suppl. Figures 2 and 3). These experiments showed that the mitochondrial biogenesis marker, Peroxisome Proliferator-activated Receptor Gamma Coactivator 1-alpha (PGC1α), was significantly increased in both BAT and iWAT from *Acss1^K635Q/K635Q^* mice following CL treatment as compared to WT. Lipolytic activity in *Acss1^K635Q/K635Q^*, as assessed by Hormone Sensitive Lipase (HSL) phosphorylated at serine 563 versus total HSL, was decreased in BAT following CL treatment, while in iWAT it was significantly increased. The lipolytic proteins, Adipose Triglyceride Lipase (ATGL) and Perilipin 5 (PLIN5) did not show major changes.

While baseline levels of UCP1 were higher in *Acss1^K635Q/K635Q^* BAT, no increase was observed following CL treatment. As expected, UCP1 was barely detectable in iWAT at baseline. However, UCP1 levels were significantly increased only in WT and not in *Acss1^K635Q/K635Q^* iWAT after CL treatment. Gene expression analysis by qPCR confirmed that, in contrast to WT, *Acss1^K635Q/K635Q^* iWAT did not significantly increase *Ucp1* expression following CL treatment (Suppl. Figure 4A). The fact that iWAT was unable to upregulate UCP1 is striking and suggests an aberrant beiging response, which may contribute to the failure of *Acss1^K635Q/K635Q^* mice to maintain body temperature.

Interestingly, ACSS1 levels tended to be higher in *Acss1^K635Q/K635Q^* adipose tissues than in WT, which was not previously observed in other tissues 5, but may be an attempt to compensate for reduced ACSS1 activity, although *Acss1* levels were not significantly different by qPCR analysis, except in *Acss1^K635Q/K635Q^* BAT 24 hours post CL injection (Suppl. Figure 4B). Finally, levels of the Hydroxyacyl-CoA Dehydrogenase Alpha subunit (HADHA), part of the trifunctional multienzyme protein for FAO, was slightly higher at basal levels in BAT, with a small decrease in *Acss1^K635Q/K635Q^* iWAT 24 hours post CL injection.

We also measured acetyl-CoA and acetate in BAT and iWAT from WT and *Acss1^K635Q/K635Q^* mice to assess the substrate and product levels that result from altered ACSS1 enzymatic activity. While we found no significant differences in BAT acetyl-CoA levels with CL treatment (Figure 3C), we observed consistently lower acetyl-CoA levels in iWAT from the *Acss1^K635Q/K635Q^* mice (Figure 3D). Acetate levels were increased in both tissues in *Acss1^K635Q/K635Q^* mice, as expected (Figure 3E and 3F). These data suggest that *Acss1^K635Q/K635Q^* iWAT specifically is not able to meet the metabolic demand elicited by CL and fails to produce adequate acetyl-CoA.

### 3.4. *Acss1^K635Q/K635Q^ ex vivo* beige adipocytes display aberrant response to forskolin

**Figure 4:**
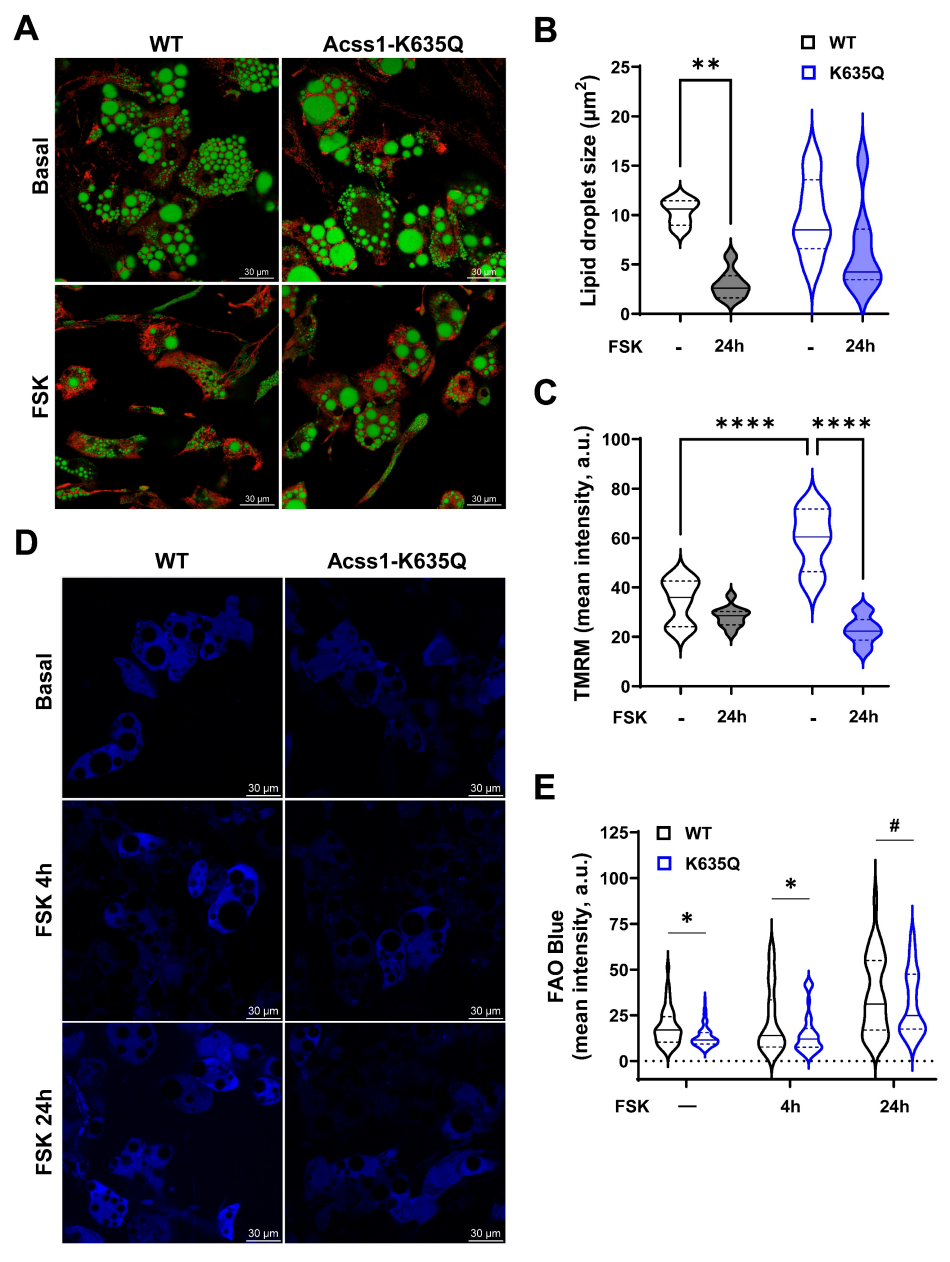
** Acss1-K635Q alters fatty acid utilization in adipocytes differentiated *ex vivo*. (A)** Adipocytes differentiated *ex vivo* from stromal cells isolated from WT and *Acss1^K635Q/K635Q^* mice were stained with bodipy for lipid droplets (green) and TMRM for mitochondrial membrane potential (red) staining ± 1 mM forskolin (FSK) for 24 h (*n* = 3 replicates). Scale bar = 30 μm. **(B-C)** Quantification of lipid droplet sizes (B) and TMRM fluorescence (C) in WT and Acss1-K635Q adipocytes ± 24 h FSK. Data distributions represented as violin plots with median and quartiles indicated. Statistical comparisons were calculated by unpaired *t*-test (**p < 0.01, ****p < 0.0001). **(D)** Adipocytes differentiated from WT and *Acss1^K635Q/K635Q^* mice stained with FAO Blue to visualize fatty acid oxidation in basal conditions and with 1 mM FSK at 4 h and 24 h (*n* = 4 replicates). Scale bar = 30 μm. **(E)** Quantification of FAO Blue fluorescence intensity at baseline and with FSK at 4 h and 24 h. Data distributions represented as violin plots with median and quartiles indicated. Statistical comparisons of WT vs. *Acss1^K635Q/K635Q^* were calculated at each timepoint by two-way ANOVA with Tukey's Multiple Comparisons test (^#^*p* < 0.1, *p < 0.05).

Stromal vascular cells were isolated from *Acss1^K635Q/K635Q^* and WT mouse iWAT and differentiated into beige adipocytes to study lipid droplet utilization and mitochondrial function. Cells were exposed to 10 μM forskolin for 24 hours in order to stimulate lipolysis and thermogenesis 27. Live cell imaging with bodipy was used to stain lipid droplets and tetramethylrhodamine methyl ester (TMRM) to assess mitochondrial membrane potential. WT beige adipocytes showed a significant reduction in lipid droplet size following forskolin treatment (Figure 4A, left panels, quantified in 4B). In contrast, *Acss1^K635Q/K635Q^* beige adipocytes showed a large range in the size of lipid droplets at baseline, which did not change with forskolin (Figure 4A, right panels, quantified in 4B). Mitochondrial membrane potential was decreased after forskolin treatment in WT cells as expected, due to UCP1 respiration uncoupling. However, *Acss1^K635Q/K635Q^* cells displayed a much higher membrane potential at baseline that was decreased following exposure to forskolin as they appeared to excessively depolarize suggesting a decrease in electron transport chain activity and the proton motive gradient (Figure 4A,C), pointing to a defect in mitochondrial function.

We used FAO Blue to measured FAO at baseline and after 4- and 24-hours forskolin treatment and found that FAO in *Acss1^K635Q/K635Q^* beige adipocytes tended to be lower than in WT at all timepoints (Figure 4D,E). All these data suggest that *Acss1^K635Q/K635Q^* beige adipocytes exhibit dysregulated lipolytic activity, FAO, and mitochondrial function.

## 4. Discussion

In this study, we demonstrate that *Acss1^K635Q/K635Q^* mice are intolerant to the thermogenic response elicited by CL being unable to maintain their body temperature with a dysregulated metabolic response. While these mice respond by increasing serum FFA, possibly increasing lipolysis to compensate for decreased FAO 21, fatty acids do not seem to be effectively oxidized. Although PGC1α is elevated in *Acss1^K635Q/K635Q^* adipose tissues and BAT showed high UCP1 levels, *Acss1^K635Q/K635Q^* iWAT failed to increase UCP1 in response to CL, suggesting impaired beiging. Mitochondria isolated from *Acss1^K635Q/K635Q^* BAT also showed impaired respiration. Although some compensatory responses appear to be present, such as higher FAO enzymatic expression and increased ACSS1, they are insufficient to compensate for ACSS1 functional deficiency in response to the metabolic demands induced by CL.

Following CL injection, we observed an immediate drop in glucose, which was recovered over the following 24 hours in wild-type mice but continued to fall in the *Acss1^K635Q/K635Q^* mice. It is interesting that some of the other metabolic changes were apparent at 4 hours while others are seen at 24 hours. While we don't have a definitive answer for this, one possible explanation is that the changes in metabolism following CL injection may be in a pathway and that the effects at 4 hours are early events and those later are downstream.

Others have demonstrated the essential role of mitochondrial fatty acid import and oxidation for BAT thermogenesis. For instance, carnitine palmitoyltransferase II (CPT2) is an integral protein of the inner mitochondrial membrane, required for the import of long-chain fatty acids and essential for thermogenesis and maintenance of BAT. Mice with adipose-specific knockout of *Cpt2* fail to induce *Ucp1* in BAT upon acute cold exposure or administration of CL, forskolin, or the general adrenergic agonist, isoproterenol 29, and showed structural deterioration of BAT in response to agonist treatment 20.

Loss of *Sirt3* has been previously shown to impair lipid utilization, thermoregulation, and respiration in BAT mitochondria, without affecting UCP1 expression suggesting that UCP1-mediated thermogenesis is indirectly regulated by deacetylation 13, although this study did not identify a specific SIRT3 deacetylation target for thermogenesis. This data also suggests that SIRT3, and its deacetylation targets, may be part of an axis that regulates how cells utilize fatty acids under differing metabolic states 12, 30, 31. In this regard, we clearly show that a constitutive ACSS1 acetylation mimic disrupts BAT lipid utilization and impairs the thermogenic response, as demonstrated by the increase in serum FFA and metabolic crisis in *Acss1^K635Q/K635Q^* mice after CL injection. Further studies are needed to delineate the molecular mechanisms underlying the role of ACSS1 in lipid metabolism and the thermogenic response.

*De novo* lipogenesis, the process of converting glucose into fatty acids, plays a crucial role in thermogenesis within brown adipocytes 32. This is unusual, as fatty acid synthesis and oxidation typically counteract each other in other tissues 33. To maintain homeostasis and thermogenesis in BAT, a tightly regulated balance exists between lipolysis and lipogenesis. *De novo* lipogenesis relies on acetyl-CoA as a substrate. Unlike other members of the acyl-CoA synthetase family, ACSS1-3 enzymes specifically catalyze the conversion of acetate and CoA into acetyl-CoA, playing a unique role in lipid metabolism. Furthermore, ACSS1 is more highly abundant in BAT as compared to WAT, suggesting a possible involvement in thermogenesis 34. In this regard, the reduced enzymatic activity of acetylated ACSS1 may lead to lower levels of acetyl-CoA and intermediates feeding the TCA cycle and ultimately to lower FAO 35. This may underlie the apparent metabolic crisis in* Acss1^K635Q/K635Q^* mice, which are unable to meet the acute energy demand elicited by CL.

Lower acetyl-CoA levels should signal for increased FAO activity and lipolysis 36, which fits our observations of increased levels of HADHA and lipolytic activity in *Acss1^K635Q/K635Q^*. Another plausible mechanism for the lower FAO activity observed in these mice might be that lower acetyl-CoA levels leads to lower fatty acid esterification with acetyl-CoA which is needed in order to activate them into acyl-CoA molecules and enter FAO cycles 37. Additionally, it has been described before that excess FFA in BAT upregulate both expression of UCP1 38. We conclude that full ACSS1 functionality is needed during the thermogenic response elicited by CL, which it is unable to meet in an acetylated state. *Acss1^K635Q/K635Q^* mice represent a constitutively acetylated enzyme that cannot be further regulated by this post-translational modification. This might lead to lower acetyl-CoA intermediates that feed the TCA cycle, causing an acute energetic deficit and failure to maintain body temperature. However, further mechanistic studies are needed to dissect the exact molecular mechanism.

## Supplementary Material

Supplementary figures.

## Figures and Tables

**Table 1:** Primary antibodies.

Antibody	Source	Identifier
ACSS1 rabbit polyclonal	Proteintech	#17138-1AP
ATGL rabbit polyclonal	Proteintech	#55190-1-AP
HADHA / Kv4.2 (aa 501-630 of hKv4.2) rabbit polyclonal	NOVUS	#NBP3-03743
HSL rabbit	Cell Signaling	#4107T
Phospho-HSL (Ser563) rabbit	Cell Signaling	#4139T
PGC1α (3G6) rabbit	Cell Signaling	#2178T
PLIN5 mouse monoclonal	Progen	#651176
UCP1 (aa 100-200 of hUCP1) synthetic polyclonal	Abcam	#ab10983
Beta Actin mouse monoclonal HRP-conjugated	Proteintech	#HRP-66009

**Table 2:** Quantitative real-time polymerase chain reaction primer sequences.

Gene (Mouse)	Forward Sequence (5' to 3')	Reverse Sequence (5' to 3')
18s	GTAACCCGTTGAACCCCATT	CCATCCAATCGGTAGTAGCG
Acss1	ACCCTGATGCTGGTCGTTAC	CGTGGTTGATAGGCTCTCCC
Ucp1	ACTGCCACACCTCCAGTCATT	CTTTGCCTCACTCAGGATTGG

## Abbreviations

ACSS1: Acyl-CoA synthetase short chain family member 1
BAT: brown adipose tissue
BCA: bicinchoninic acid
CL: CL-316243
CoA: coenzyme A
DMEM: Dulbecco's modified Eagle medium
EGTA: ethylene glycol tetraacetic acid
FAO: fatty acid oxidation
FFA: free fatty acid
FSK: forskalin
G3P: glycerol-3-phosphate
GDP: guanosine diphosphate
H&E: haematoxylin and eosin
HADHA: Hydroxyacyl-CoA Dehydrogenase Alpha subunit
HEPES: 4-(2-hydroxyethyl)-1-piperazineethanesulfonic acid
HRP: horseradish peroxidase
HSL: hormone sensitive lipase
IP: intra-peritoneal
iWAT: inguinal white adipose tissue
MAS: mitochondria assay solution
OCR: oxygen consumption rate
PBS: phosphate buffered saline
PGC1α: peroxisome proliferator-activated receptor gamma coactivator 1-alpha
PVDF: polyvinylidene difluoride
SDS-PAGE: sodium dodecyl sulfate polyacrylamide gel electrophoresis
SIRT3: sirtuin 3
TMRM: tetramethylrhodamine methyl ester
UCP1: uncoupling protein 1
WAT: white adipose tissue
WT: wild-type
