# Supplementary material for: β3-adrenergic receptor agonist causes acute thermogenic metabolic crisis in ACSS1-K635Q knock-in mice
Source: Int J Biol Sci. 2026 Jan 1;22(3):1070–81. doi: 10.7150/ijbs.122643 (PMC12836529; doi:10.7150/ijbs.122643)
Supplement: Supplementary file 1 — Supplementary figures. [file ijbsv22p1070s1.pdf]

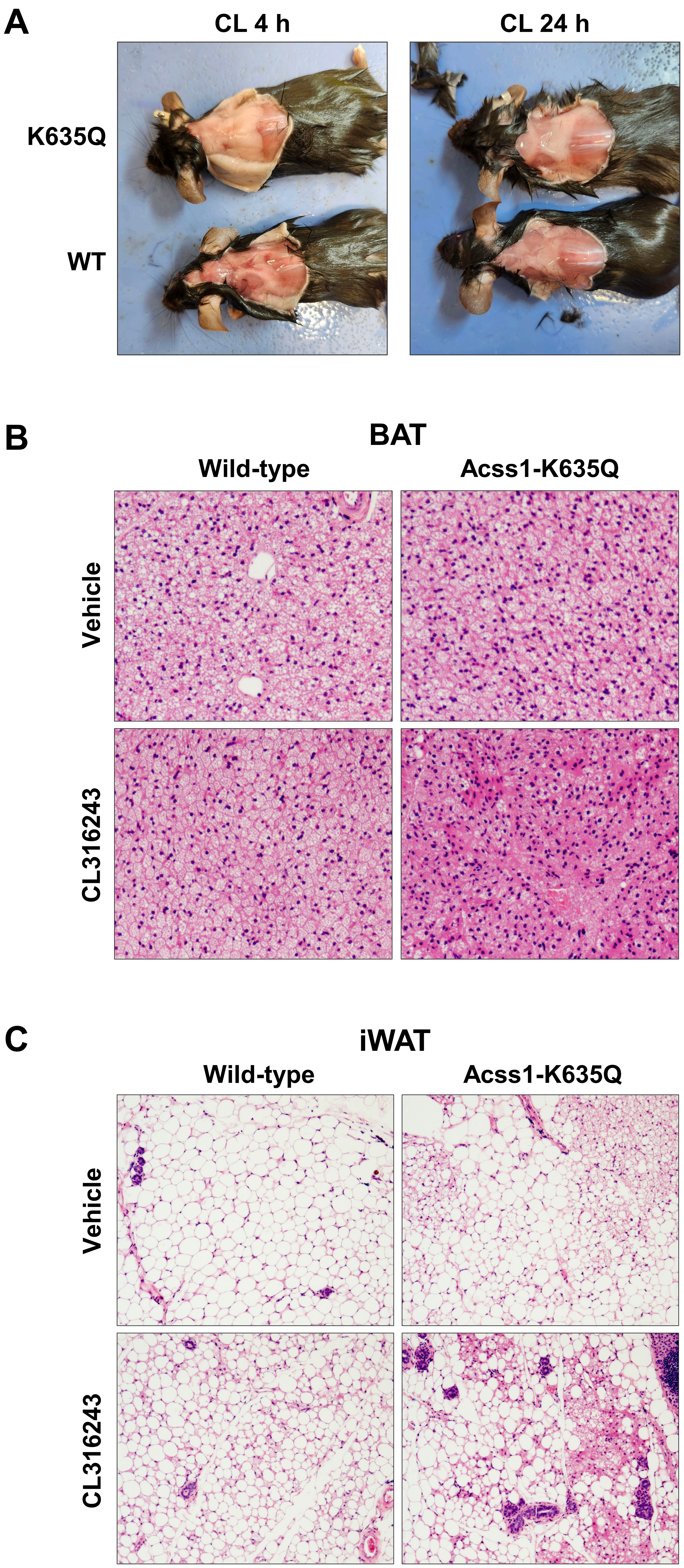

Figure S1. Adipose histology in wild-type vs. *Acss1*$^{K635Q/K635Q}$ mice.
(A) Suprascapular WAT pictures 4- and 24-hours post CL injection in 3-month-old female WT and *Acss1*$^{K635Q/K635Q}$ mice (n = 2 mice per group).
(B) H&E staining of BAT collected from WT (left column) and *Acss1*$^{K635Q/K635Q}$ (right column) mice 24 hours after injection of vehicle (top row) or CL (bottom row), shown at 10x magnification (n = 3 mice per group).
(C) H&E staining of iWAT collected from WT (left column) and *Acss1*$^{K635Q/K635Q}$ (right column) mice 24 hours after injection of vehicle (top row) or CL (bottom row), shown at 10x magnification (n = 3 mice per group).

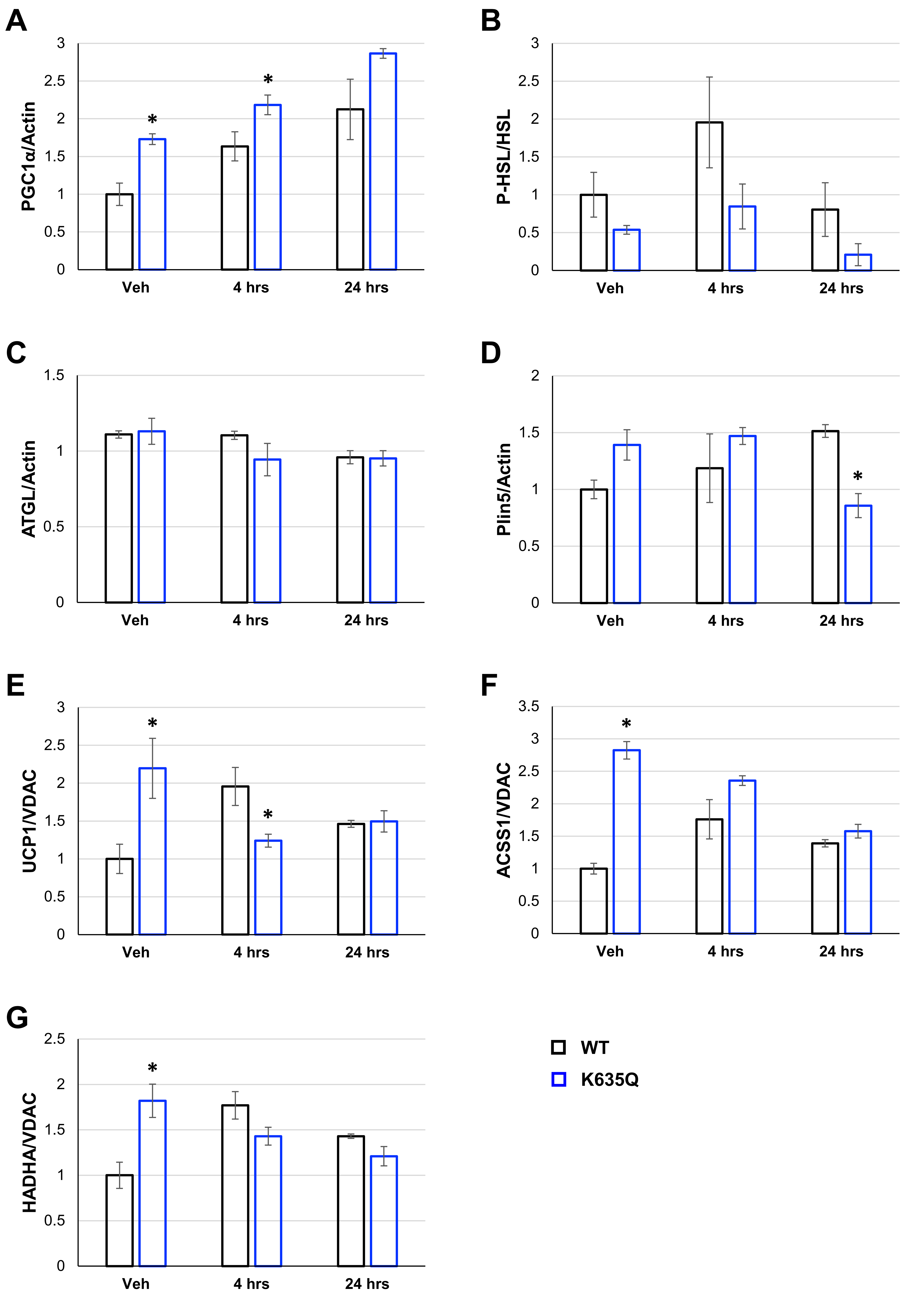

Figure S2. BAT protein levels in wild-type vs. *Acss1*$^{K635Q/K635Q}$ mice following CL injection.
(A-G) Quantification of PGC1α (A) relative to actin, HSL phosphorylated at serine 563 relative to total HSL (B), ATGL (C) and PLIN5 (D) relative to actin, and UCP1 (E), ACSS1 (F) and HADHA (G) relative to VDAC in BAT immunoblot (Figure 3A), using ImageJ band densitometry. Data shown as mean ± SEM (n = 3 mice for vehicle and 4 per treatment group). Comparisons were done by unpaired t-tests (*$p < 0.05$).

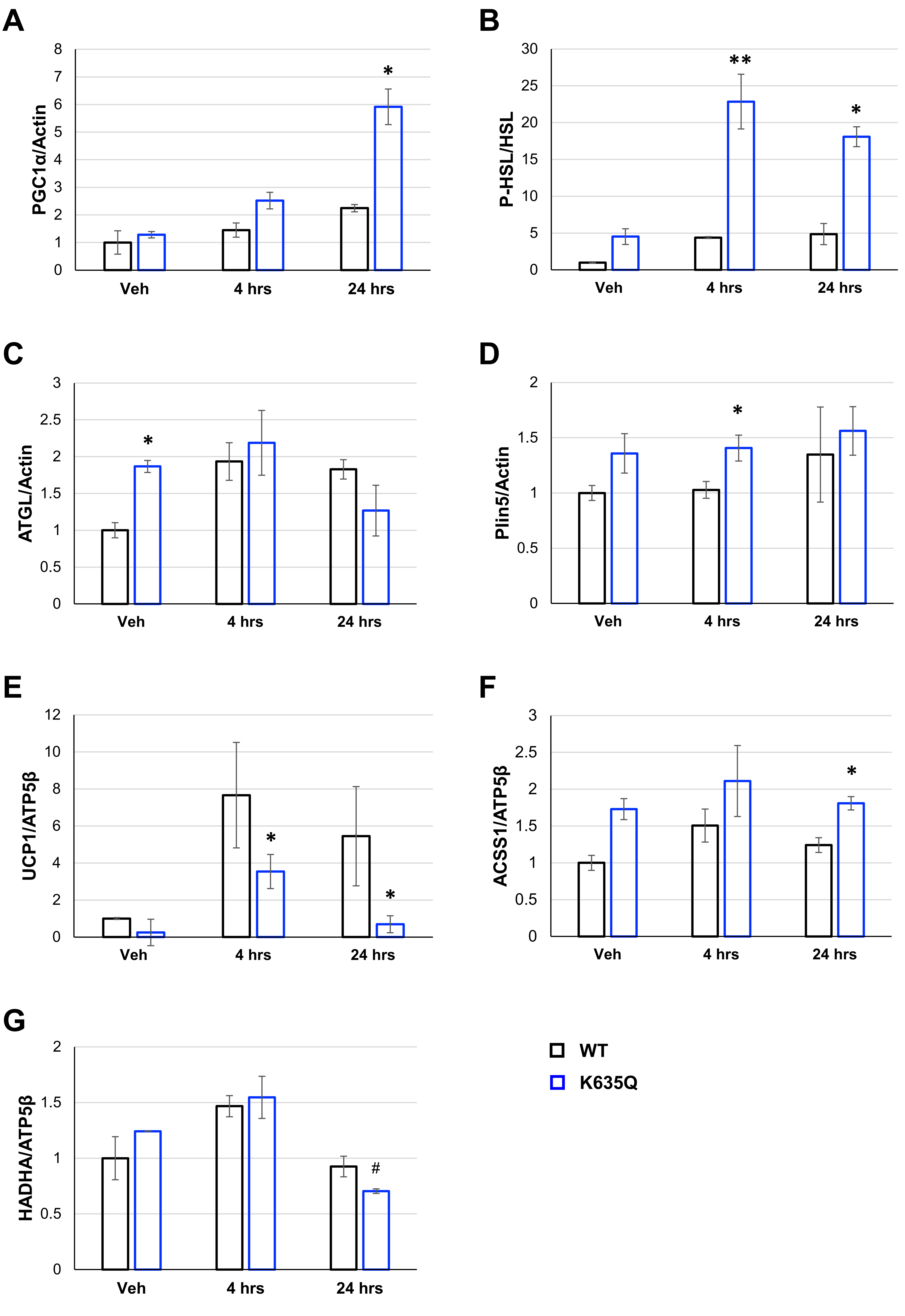

Figure S3. iWAT protein levels in wild-type vs. *Acss1*$^{K635Q/K635Q}$ mice following CL injection.
(A-G) Quantification of PGC1α (A) relative to actin, HSL phosphorylated at serine 563 relative to total HSL (B), ATGL (C) and PLIN5 (D) relative to actin, and UCP1 (E), ACSS1 (F) and HADHA (G) relative to ATP5β in iWAT immunoblot (Figure 3B), using ImageJ band densitometry. Data shown as mean ± SEM (n = 3 mice for vehicle and 4 per treatment group). Comparisons were done by unpaired t-tests (#$p < 0.1$, *$p < 0.05$, **$p < 0.01$).

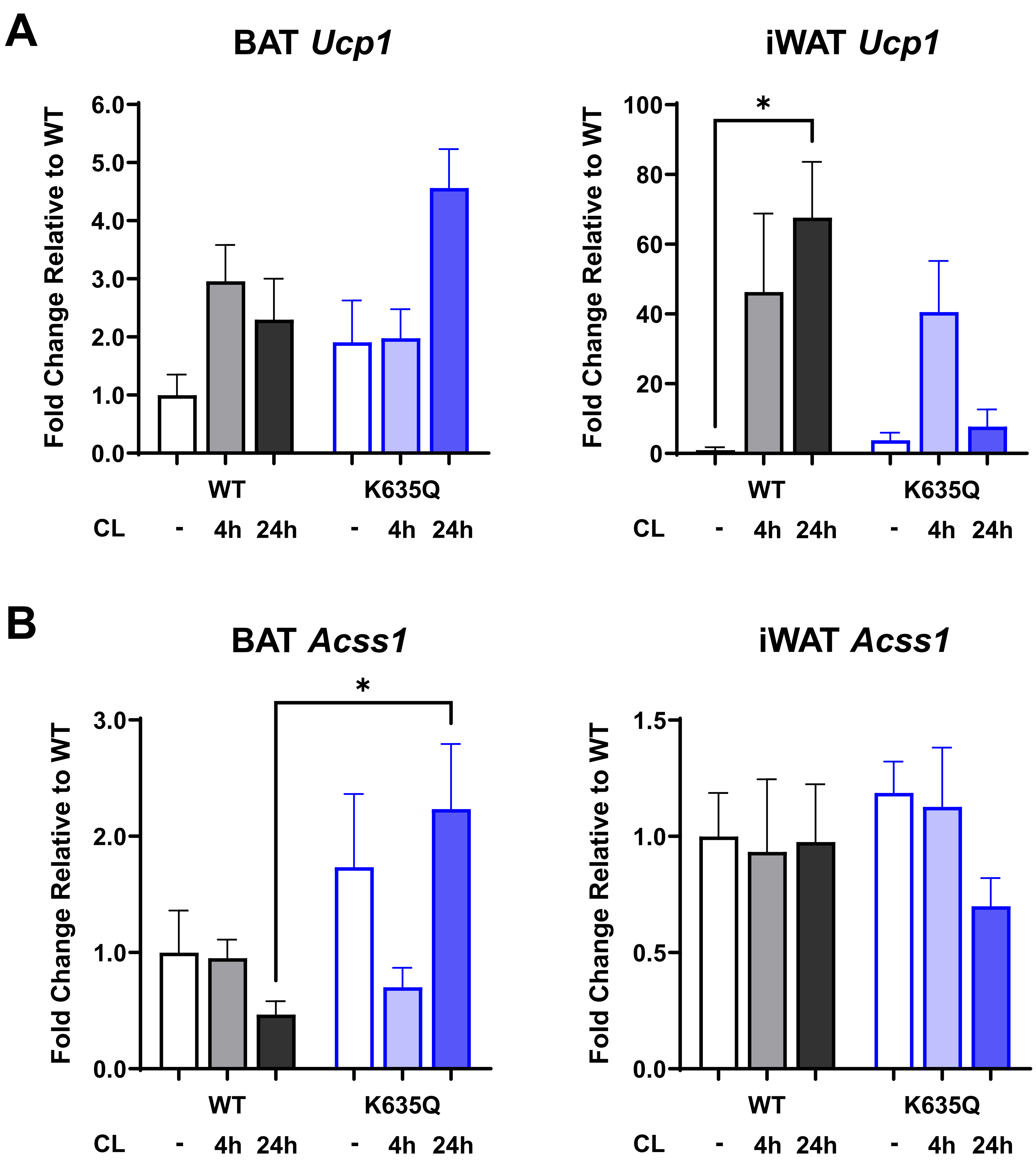

Figure S4. Gene expression in wild-type vs. *Acss1*$^{K635Q/K635Q}$ mice following CL injection.
(A) Quantitative PCR of *Ucp1* expression levels in BAT and iWAT from WT and *Acss1*$^{K635Q/K635Q}$ mice following injection of vehicle or 4h or 24h after CL injection (n = 3 mice for vehicle and 4 per treatment group).
(B) Quantitative PCR of *Acss1* expression levels in BAT and iWAT from WT and *Acss1*$^{K635Q/K635Q}$ mice following injection of vehicle or 4h or 24h after CL injection (n = 3 mice for vehicle and 4 per treatment group).
Gene expression levels were quantified by the $2^{-\Delta\Delta CT}$ method, using the 18S gene as a normalizing control. Data represented as mean ± SEM. Comparisons were done by one-way ANOVA (*$p < 0.05$)
